# Genetic diversity of *Phlebotomus perniciosus* populations between insular and mainland regions in the leishmaniasis-endemic western Mediterranean area

**DOI:** 10.1186/s13071-026-07261-z

**Published:** 2026-01-22

**Authors:** Sarah Chavez-Fisa, Xavier Roca-Geronès, Roser Fisa, Cristina Riera, M. Magdalena Alcover

**Affiliations:** Departament de Biologia, Sanitat i Medi Ambient. Secció de Parasitologia, Facultat de Farmàcia i Ciències de l’Alimentació. Universitat de Barcelona, Barcelona, Spain

**Keywords:** *Phlebotomus perniciosus*, Majorca, Barcelona, Iberian Peninsula, North Africa, Insularity, Population genetics, Mitochondrial cytochrome c oxidase subunit I

## Abstract

**Background:**

*Phlebotomus perniciosus* is the primary vector of *Leishmania infantum* in Spain, occurring in both continental and insular regions. This study investigates the genetic structure of *P. perniciosus* populations from Majorca (island) and Barcelona (mainland), two geographically close but ecologically distinct regions in the western Mediterranean.

**Methods:**

Mitochondrial cytochrome c oxidase subunit I (COI) gene sequences were analyzed from 167 *P. perniciosus* specimens, including 100 morphologically identified field-collected specimens from Majorca and Barcelona, supplemented with reference data from the South and West Iberian Peninsula and North Africa. Population differentiation was assessed using genetic diversity indices, Bayesian phylogenetic inference, analysis of molecular variance (AMOVA), pairwise *Fst* values and *Nm* estimates, haplotype networks, and a Mantel test.

**Results:**

Phylogenetic analysis confirmed the morphological identification of all *P. perniciosus* specimens, grouping them into a single clade, with distinct subclades corresponding to the geographical origin. Haplotype analysis revealed 56 genetic variants, with the predominant haplotype represented by 37 specimens in Majorca and 40 in Barcelona. Significant genetic differentiation was observed between populations from Majorca and Barcelona (*Fst* = 0.78262, *P* < 0.00001), indicating limited gene flow. Nucleotide diversity was higher in Majorca (*π* ± *SD* = 0.0037 ± 0.00090) than in Barcelona (*π* ± *SD* = 0.0006 ± 0.00021). Majorcan specimens showed close genetic affinity to the Algerian and Tunisian populations (*Fst* = 0.02470, *P* > 0.05), while Barcelona specimens were more closely related to those of the South and West Iberian Peninsula (*Fst* = 0.51225, *P* < 0.00001).

**Conclusions:**

These findings indicate that geographic isolation and historical dispersal may have shaped the *P. perniciosus* genetic structure. The Balearic Sea appears to act as a significant barrier, restricting gene flow between island and Iberian mainland populations. The study supports the utility of COI in phylogeographic research and demonstrates how island–mainland comparisons can help reveal evolutionary processes in vector species.

**Graphical Abstract:**

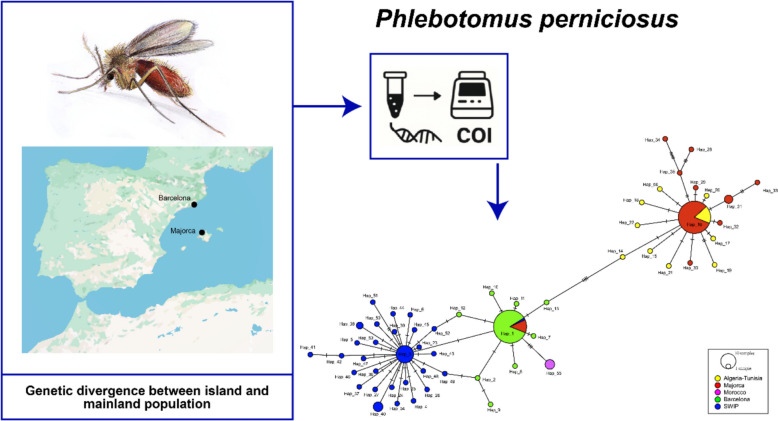

**Supplementary Information:**

The online version contains supplementary material available at 10.1186/s13071-026-07261-z.

## Background

Phlebotomine sand flies (Diptera: Psychodidae: Phlebotominae) are hematophagous insects found in tropical, subtropical, and temperate regions. They are of major medical and veterinary importance as vectors of bacterial, viral, and protozoan pathogens. These include kinetoplastid parasites of the genus *Leishmania*, the causative agents of leishmaniasis in humans and approximately 70 other animal species worldwide [1, 2]. The World Health Organization (WHO) lists human leishmaniasis among the top ten neglected tropical diseases, with an estimated 700,000 to 1 million new cases each year [2]. In Europe, leishmaniasis is an emerging zoonosis, with around 700 autochthonous human cases reported annually in southern countries, although the true incidence is likely higher owing to underreporting [3].

*Leishmania infantum* is widespread in the Iberian Peninsula and the Balearic Islands, where it causes visceral (VL), cutaneous (CL), and mucocutaneous (MCL) forms of leishmaniasis in humans and is also the agent of canine leishmaniasis [4, 5]. Within the Mediterranean basin, *L. tropica* and *L. major* are responsible for CL in North Africa and the Middle East. *L. tropica* has also been reported in Greece, Turkey, Serbia, and Cyprus [6, 7].

In Spain, human leishmaniasis is an endemic and notifiable disease. Between 2019 and 2021, 1039 confirmed cases were reported across 16 autonomous communities. Incidence was highest in the Valencian Community (2.98/100,000 inhabitants) and the Balearic Islands (2.97/100,000 inhabitants), while Catalonia (where Barcelona is located) reported a lower rate (0.59/100,000 inhabitants) [8]. However, underreporting remains a significant concern, particularly for CL [8]. In the Balearic Islands, cases of human leishmaniasis have been documented since 1925, with Majorca consistently exhibiting the highest incidence in the archipelago [9, 10].

In the Old World, *Phlebotomus* species are the primary vectors of *Leishmania*, whereas in the Americas, transmission is mediated by *Lutzomyia* species [11]. A close ecological, and possibly co-evolutionary, relationship exists between *Leishmania* species and their specific vectors [12]. In total, 23 sand fly species are known in Europe, belonging to the genera *Phlebotomus* and *Sergentomyia* [13], 13 of which occur in Spain [14]. In the Balearic Islands, four species have been recorded: *P. perniciosus*, *P. sergenti*, *P. papatasi*, and *S. minuta* [15]. Recently, *P. perfiliewi*, a known vector of *L. infantum*, was detected in Majorca [16], expanding the documented diversity of sand flies in the region. *P. perfiliewi* is considered a competent vector in the Mediterranean Basin, with confirmed involvement in Italy, Greece, and Tunisia, and its detection in Majorca and other areas of Southern Europe and North Africa highlights the need to assess its potential role in local transmission dynamics [17–19].

In Spain, *P. perniciosus* and *P. ariasi* are the main vectors, with the former predominating in both Majorca and the Barcelona area [14, 15]. *P. ariasi* has a more focal distribution in Spain, being mainly associated with cooler and more humid mountainous areas [20]. In contrast, *P. perniciosus* is widely distributed across the country [14] and exhibits high ecological plasticity, thriving in climates ranging from subhumid to arid conditions. It is the dominant sand fly in many endemic regions, except in those cooler, more humid zones where *P. ariasi* predominates. In Spain, the seasonal activity of *P. perniciosus* typically extends from May to November, peaking during the early nighttime hours [15, 20, 21].

Genetic tools have become increasingly important in the study of sand fly diversity and phylogeography. Molecular markers such as isoenzymes, microsatellites, the internal transcribed spacer 2 (ITS2), and mitochondrial genes—including cytochrome b (Cyt b), cytochrome c oxidase subunit I (COI), and NADH dehydrogenase subunit 4 (ND4)—have been widely used to assess genetic diversity and population structure in multiple *Phlebotomus* and *Lutzomyia* species [21–27]. These approaches provide valuable insights into evolutionary relationships, dispersal patterns, and potential barriers to gene flow, all of which could influence vector competence, host preference, and disease transmission, thereby supporting more effective vector surveillance and targeted control strategies. Among mitochondrial markers, COI is particularly valuable in population genetics and phylogeography owing to its maternal inheritance, absence of recombination, conserved primer-binding regions, and a relatively high mutation rate [28]. Consequently, it has been successfully applied to resolve species boundaries, population structure, and genetic diversity in sand flies [29–31].

Islands can be considered as natural laboratories for evolutionary studies. Their isolation and defined boundaries provide simplified contexts for observing processes such as colonization, extinction, and potentially, speciation, offering clearer insights than more interconnected mainland ecosystems [32–34]. Comparing genetic patterns between island and mainland populations allows for a deeper understanding of the ecological and evolutionary forces shaping biodiversity [33].

In the present study, the genetic diversity and population structure of *P. perniciosus* from Majorca and Barcelona were analyzed using the mitochondrial COI marker. By comparing these insular and mainland populations, this work aimed to determine the extent to which geographic isolation influences the genetic variability and structure of this medically important leishmaniasis vector, with potential implications for disease transmission and vector control.

## Methods

### Sand fly collection and morphological identification

Sand flies were collected during the summer months (June to September) of 2023 and 2024 in two distinct geographic regions: the island of Majorca and the area surrounding Barcelona. In Majorca, collections were carried out at three rural locations: two sites in the municipality of Montuïri (a) 39° 33′ 44.0″ N, 3° 01′ 08.5″ E; (b) 39° 33′ 42.9″ N, 3° 01′ 07.8″ E; and one site in the municipality of Sant Joan (c) 39° 34′ 27.4″ N, 3° 01′ 30.4″ E. In the Barcelona area, sampling was conducted at three rural sites near the city: two located in the municipality of Sant Just Desvern (a) 41° 23′ 52.6″ N, 2° 05′ 05.0″ E; (b) 41° 23′ 22.5″ N, 2° 04′ 17.7″ E; and one in the municipality of Barcelona (c) 41° 26′ 03.3″ N, 2° 07′ 55.1″ E.

Collections were performed using CDC light traps, strategically placed in both domestic and peridomestic environments to maximize capture efficiency. Traps were operated overnight, from dusk until dawn, coinciding with the known peak activity period of phlebotomine sand flies.

Collected specimens were preserved immediately in 70% ethanol to maintain both morphological integrity and DNA quality. Sand flies were sexed and identified to species level under a stereomicroscope using the standard taxonomic keys of Gállego et al. [35]. Male genitalia were dissected and mounted in Hoyer’s medium for microscopic analysis. For females, the head and the last three abdominal segments were similarly dissected and mounted for taxonomic identification. Remaining body parts were stored at −20 °C for molecular analysis.

### DNA extraction and PCR amplification

Genomic DNA was individually extracted from morphologically identified *P. perniciosus* specimens using a Chelex^®^ 100 resin-based protocol as described by Tomás-Pérez et al. [36]. A 658-base pair (bp) fragment of the mitochondrial COI gene was amplified by polymerase chain reaction (PCR) using the universal primers LCO1490 (5′–GGTCAACAAATCATAAAGATATTGG–3′) and HCO2198 (5′–TAAACTTCAGGGTGACCAAAAAATCA–3′) [37].

PCRs were performed in a thermocycler following the protocol of Hebert et al. [28]: initial denaturation at 94 °C for 3 min; 35 cycles of denaturation at 94 °C for 30 s, annealing at 55 °C for 1 min, and 72 °C for 1 min; and a final extension at 72 °C for 10 min. Amplification products were verified by agarose gel electrophoresis (1%) and visualization under UV light. A 100 bp ladder (EZ Load, Bio-Rad) served as the molecular weight marker.

### Sequencing and sequence analysis

PCR products were sequenced bidirectionally using the same primers at the Genomics Unit of the Scientific and Technological Centers of the University of Barcelona (CCiTUB). Sequence chromatograms were checked using Sequence Scanner Software v.2 to evaluate signal quality, trim low-quality ends, and confirm base-calling accuracy. Forward and reverse reads were manually inspected for ambiguities and assembled into consensus sequences. Sequences with poor signal quality were excluded to ensure data reliability. Multiple sequence alignments were performed using the ClustalW algorithm implemented in MEGA v.11 [38]. Publicly available COI sequences of *P. perniciosus* and related species were retrieved from GenBank to complement the dataset and support phylogenetic inference.

The best-fitting nucleotide substitution model was selected using the Akaike information criterion (AIC) in jModelTest v.2.1.10 [39]. Phylogenetic relationships were inferred using Bayesian inference in MrBayes v.3.2.7 [40], with analyses run for 1,000,000 generations and trees sampled every 100 generations. Nodes with posterior probabilities (PP) ≥ 0.90 were considered strongly supported. The final phylogenetic tree included *Phlebotomus* species from diverse geographic locations (Table 1).

**Table 1:** List of COI sequences used for the Bayesian inference phylogenetic analysis, including *P. perniciosus* specimens from the present study (Majorca and Barcelona) and sequences of *Phlebotomus* species and *S. minuta* (outgroup) retrieved from GenBank, with their corresponding accession numbers, geographic origins, and references

*Phlebotomus* species	Genbank accession number	Geographical origin	Reference
*P. perniciosus*	PV672187–90	Majorca	Present study
*P. perniciosus*	PV672191–94	Barcelona	Present study
*P. perniciosus*	OR076248	South Spain	[70]
*P. perniciosus*	OR076182	South Spain	[70]
*P. perniciosus*	AB985703	Portugal	[71]
*P. perniciosus*	AB985717	Portugal	[71]
*P. perniciosus*	KJ481140	Algeria	[72]
*P. perniciosus*	KJ481151	Algeria	[72]
*P. perniciosus*	OL814952	Tunisia	[73]
*P. perniciosus*	OL814955	Tunisia	[73]
*P. perniciosus*	MT233398	Morocco	[74]
*P. perniciosus*	MT233399	Morocco	[74]
*P. papatasi*	PP389414	Uzbekistan	Unpublished
*P. papatasi*	MT074063	Morocco	Unpublished
*P. ariasi*	OL364754	Portugal	[75]
*P. ariasi*	OL364747	Portugal	[75]
*P. sergenti*	MZ049666	Egypt	[55]
*P. sergenti*	OL352173	Greece	[76]
*P. longicuspis*	MT237931	Morocco	[74]
*P. longicuspis*	MT240847	Morocco	[74]
*P. perfiliewi*	OP824890	Majorca	[16]
*P. perfiliewi*	OP169333	Jordan	Unpublished
*S. minuta*	MT240854	Morocco	[74]

### Intraspecific genetic diversity and population structure

Intraspecific genetic diversity of *P. perniciosus* populations sampled in Majorca and Barcelona, inferred from COI sequence analysis, was compared with 67 additional COI sequences retrieved from GenBank, representing conspecific populations from Algeria, Tunisia, Majorca, southern Spain, Portugal, and Morocco (Supplementary Table 1).

Genetic diversity parameters were estimated using DnaSP v.6 [41], including the number of haplotypes (*Nh*) and unique/private haplotypes (*Nuh*), nucleotide diversity (*π*), haplotype diversity (*Hd*), average number of nucleotide differences (*K*), and the number of segregating sites (*S*).

Estimation of genetic differentiation between populations was performed using Wright’s fixation index (*Fst*), estimated as *Φst* for sequence data [42, 43]. Analyses were conducted in Arlequin v.3.5 with 1000 permutations, and the threshold of significance was set at *P* < 0.05 [44]. Following the criterion for genetic differentiation from Wright [43], *Fst* values from 0 to 0.05 were considered indicative of little or negligible differentiation between populations, values from 0.05 to 0.15 were interpreted as moderate differentiation, values from 0.15 to 0.25 as high differentiation, and values above 0.25 as very high differentiation. Spatial analysis of molecular variance (AMOVA) was performed using a three-level hierarchical design [45]. Variance was partitioned among groups, among populations within groups, and within populations, with significance tested by 1000 permutations. To further assess genetic connectivity among populations, the number of migrants per generation (*Nm*) was estimated from pairwise *Fst* values following the infinite-island model of population structure and gene flow [46]. Values of *Nm* below 1 were interpreted as indicative of restricted gene flow and substantial population differentiation. To examine whether any isolation-by-distance (IBD) effect occurred, a Mantel test was performed in Arlequin v.3.5, comparing pairwise *Fst* values with the logarithm of geographical distances between sampling populations [47]. Because exact capture coordinates were not available, distances were estimated from the nearest documented localities.

To visualize haplotype relationships and infer potential ancestral connections, a haplotype network was constructed on the basis of statistical parsimony using the TCS algorithm implemented in PopART v.1.7 [48]. The analysis followed the 95% parsimony connection limit criterion implemented in TCS [49]. Circle sizes in the network are proportional to haplotype frequencies.

## Results

### Sequence analysis

A total of 125 *P. perniciosus* specimens were morphologically identified and processed for molecular analysis. All samples yielded successful PCR amplification, and 100 (80%) produced high-quality bidirectional COI sequences that were included in downstream analyses. Of these successfully sequenced specimens, 52 originated from Majorca and 48 from Barcelona. Newly generated COI region sequences were deposited in GenBank under accession numbers PV672187–94. After alignment and trimming to exclude ambiguous sites, the final dataset consisted of 574 nucleotide positions. Sequence alignment of the COI region did not reveal any nucleotide insertions or deletions.

### Phylogenetic analysis

**Fig. 1:**
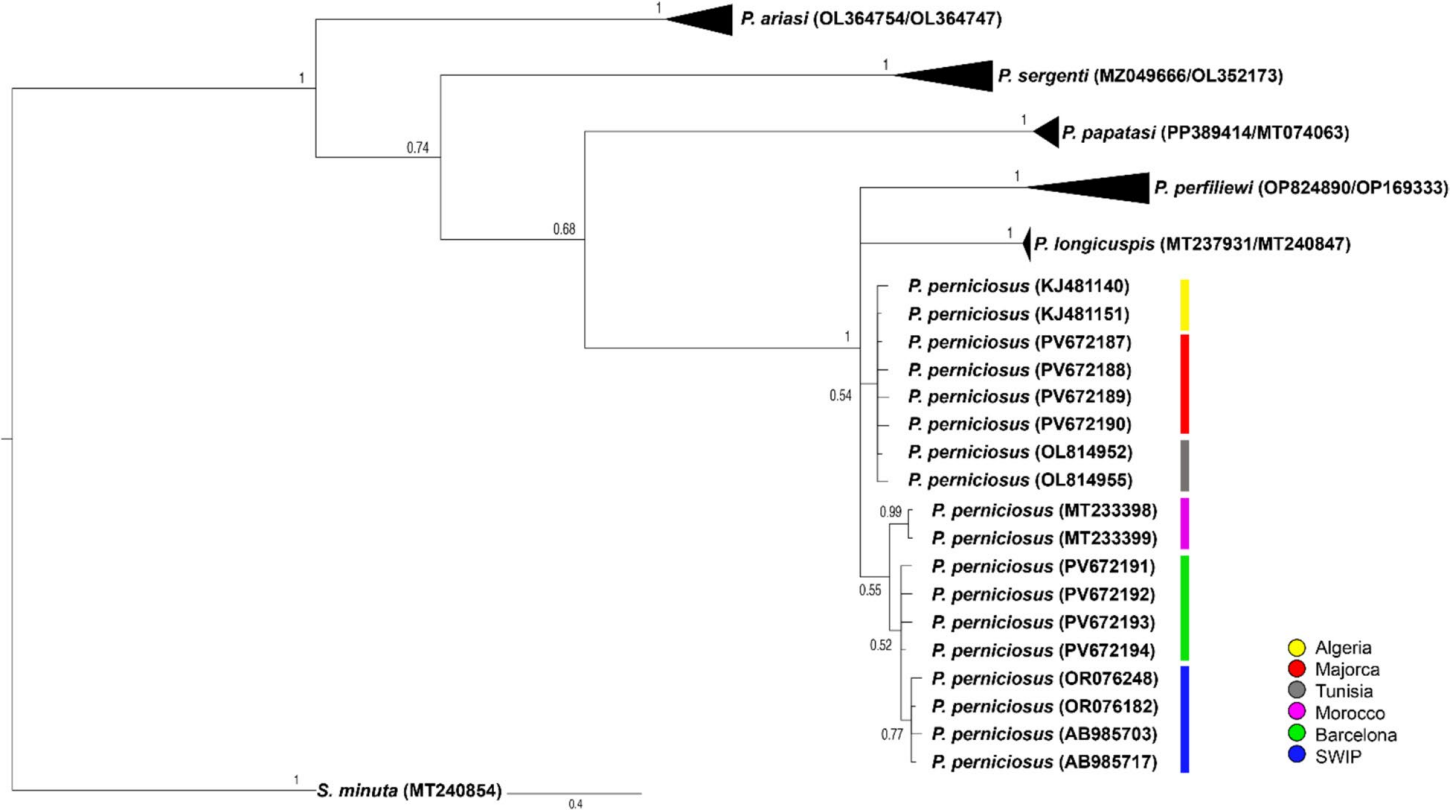
Bayesian inference phylogenetic tree of *P. perniciosus* based on COI gene sequences obtained in the present study (Majorca and Barcelona), compared with other *Phlebotomus* spp. sequences from GenBank. *S. minuta* was used as an outgroup. Color bars indicate the geographic origin of each sequence. SWIP, South and West Iberian Peninsula

The best-fit substitution model for the obtained sequences was GTR + I (*I* = 0.7209). Bayesian inference produced a phylogenetic tree in which clustering patterns reflected geographical origin, although several nodes showed moderate or low posterior probabilities (Fig. 1). Specimens from Majorca formed a distinct branch, separate from other Mediterranean populations, and showed the closest genetic affinity to Algerian and Tunisian samples. In contrast, samples from Barcelona clustered with populations from Morocco, southern Spain, and Portugal. A moderately supported subclade comprised similar sequences from southern Spain and Portugal. The analysis resolved the *P. perniciosus* group as phylogenetically distinct from other *Phlebotomus* species.

### Population differentiation

As *Fst* estimates (Table 2) showed no significant genetic differentiation between populations from southern Spain and Portugal (*Fst* = 0.00963, *P* > 0.05), these were pooled into a single “South and West Iberian Peninsula” (SWIP) group. Similarly, no significant differentiation was detected between Algerian and Tunisian populations (*Fst* = −0.07252, *P* > 0.05), which were therefore pooled into a single “Algeria–Tunisia” group. In contrast, the Majorca and Barcelona populations were highly differentiated (*Fst* = 0.78262, *P* < 0.00001) suggesting limited gene flow. The Majorcan population exhibited low differentiation from the Algeria–Tunisia group (*Fst* = 0.02470, *P* > 0.05), whereas Barcelona was significantly differentiated from the SWIP group (*Fst* = 0.51225, *P* < 0.00001). Morocco appeared genetically distinct from all other regions, with *Fst* values ranging from 0.73 to 0.89 (all *P* < 0.00001), confirming very high differentiation. These population groupings (SWIP and Algeria–Tunisia) were consistently applied in all subsequent analyses, including the estimation of *Nm*, the Mantel test, and the hierarchical AMOVA. *Nm* estimates indicated a high apparent connectivity between Majorca and the Algeria–Tunisia group (*Nm* = 19.74) (Supplementary Table 2), whereas gene flow among the remaining populations was limited (*Nm* < 1). The Mantel test revealed a low correlation between genetic and geographic distances (*R* = 0.1919, *P* = 0.2160), suggesting that genetic differentiation among populations was not significantly explained by geographic separation.

**Table 2:** Pairwise *Fst* estimates among *P. perniciosus* populations from the studied geographical origins based on COI gene sequences

	Majorca	Barcelona	SWIP	Algeria–Tunisia	Morocco
Majorca		0.00000 ± 0.0000*	0.00000 ± 0.0000*	0.11035 ± 0.0096	0.00000 ± 0.0000*
Barcelona	0.78262		0.00000 ± 0.0000*	0.00000 ± 0.0000*	0.00000 ± 0.0000*
SWIP	0.75375	0.51225		0.00000 ± 0.0000*	0.00000 ± 0.0000*
Algeria–Tunisia	0.02470	0.91029	0.82800		0.00098 ± 0.0010*
Morocco	0.73800	0.89395	0.73030	0.87415	

SWIP, South and West Iberian Peninsula. *Statistically significant (*P* < 0.05). Below the diagonal: pairwise *Fst* values; above the diagonal: corresponding *P*-values

Spatial analysis of molecular variance (AMOVA) was performed using a three-level hierarchical design to quantify the distribution of genetic variation among predefined geographical groupings, among populations within groups, and within populations. Populations were assigned to two broad geographic groups: group 1 (Majorca + Algeria–Tunisia) and group 2 (Barcelona + SWIP + Morocco). AMOVA revealed that the largest proportion of genetic variation occurred among groups (67.57%), indicating strong partitioning between these two regional groupings. A further 13.10% of the variation was attributed to differences among populations within groups, while 19.33% occurred within populations (Table 3).

**Table 3:** Three-level hierarchical AMOVA of *P. perniciosus* COI sequences, showing the partitioning of genetic variation among groups, among populations within groups, and within populations, with corresponding fixation indices

Source of variation	^a^d.f.	Sum of squares	Variance components	Variation (%)
Among groups	1	207.750	2.30256 Va	67.57
Among populations within groups	3	36.776	0.44631 Vb	13.10
Within populations	162	106.694	0.65861 Vc	19.33
Total	166	351.210	3.40748	
Fixation indices				
FCT	0.67574			
FSC	0.40393			
FST	0.80672			

^a^d.f, degree of freedom. Va, Vb, and Vc correspond to the covariance components associated with variance among groups, among populations within groups, and within populations, respectively. FCT, FSC, and FST are the corresponding hierarchical F-statistics

As shown in Table 4, the SWIP population group had the highest haplotype diversity (*Hd* ± *SD* = 0.914 ± 0.036) and a large number of private haplotypes (*Nuh* = 28). Majorca showed slightly higher haplotype diversity (*Hd* ± *SD* = 0.504 ± 0.081) than Barcelona (*Hd* ± *SD* = 0.309 ± 0.088). Nucleotide diversity was moderate overall, with Majorca presenting higher values (*π* ± *SD* = 0.0037 ± 0.00090) and a greater average number of nucleotide differences (*K* = 2.139). In contrast, the Barcelona population showed lower nucleotide diversity (*π* ± *SD* = 0.0006 ± 0.00021) and a smaller average number of nucleotide differences (*K* = 0.373). The Algeria–Tunisia population displayed relatively high haplotype diversity (*Hd* ± SD = 0.794 ± 0.103) but moderate nucleotide diversity (*π* ± *SD* = 0.0020 ± 0.00045). Moroccan population was represented by only four sequences, all corresponding to a single haplotype, showing no genetic diversity.

**Table 4:** Genetic diversity of *P. perniciosus* from Majorca, Barcelona, South and West Iberian Peninsula (SWIP), and Algeria–Tunisia inferred from COI gene sequence analysis

Population	*N*	*Nh*	*Nuh*	*S*	*K*	*Hd* ± *SD*	*π* ± *SD*
Majorca	53	10	8	20	2.139	0.504 ± 0.081	0.0037 ± 0.00090
Barcelona	48	9	8	8	0.373	0.309 ± 0.088	0.0006 ± 0.00021
SWIP	45	29	28	29	1.495	0.914 ± 0.036	0.0026 ± 0.00026
Algeria–Tunisia	17	10	9	10	1.176	0.794 ± 0.103	0.0020 ± 0.00045

*N*, number of sequences analyzed; *Nh*, number of haplotypes; *Nuh*, number of private haplotypes; *S*, number of polymorphic sites; *K*, average number of nucleotide differences; *Hd*, haplotype diversity; *SD*, standard deviation; *π*, nucleotide diversity (per site); SWIP, South and West Iberian Peninsula

### Genetic diversity

**Fig. 2:**
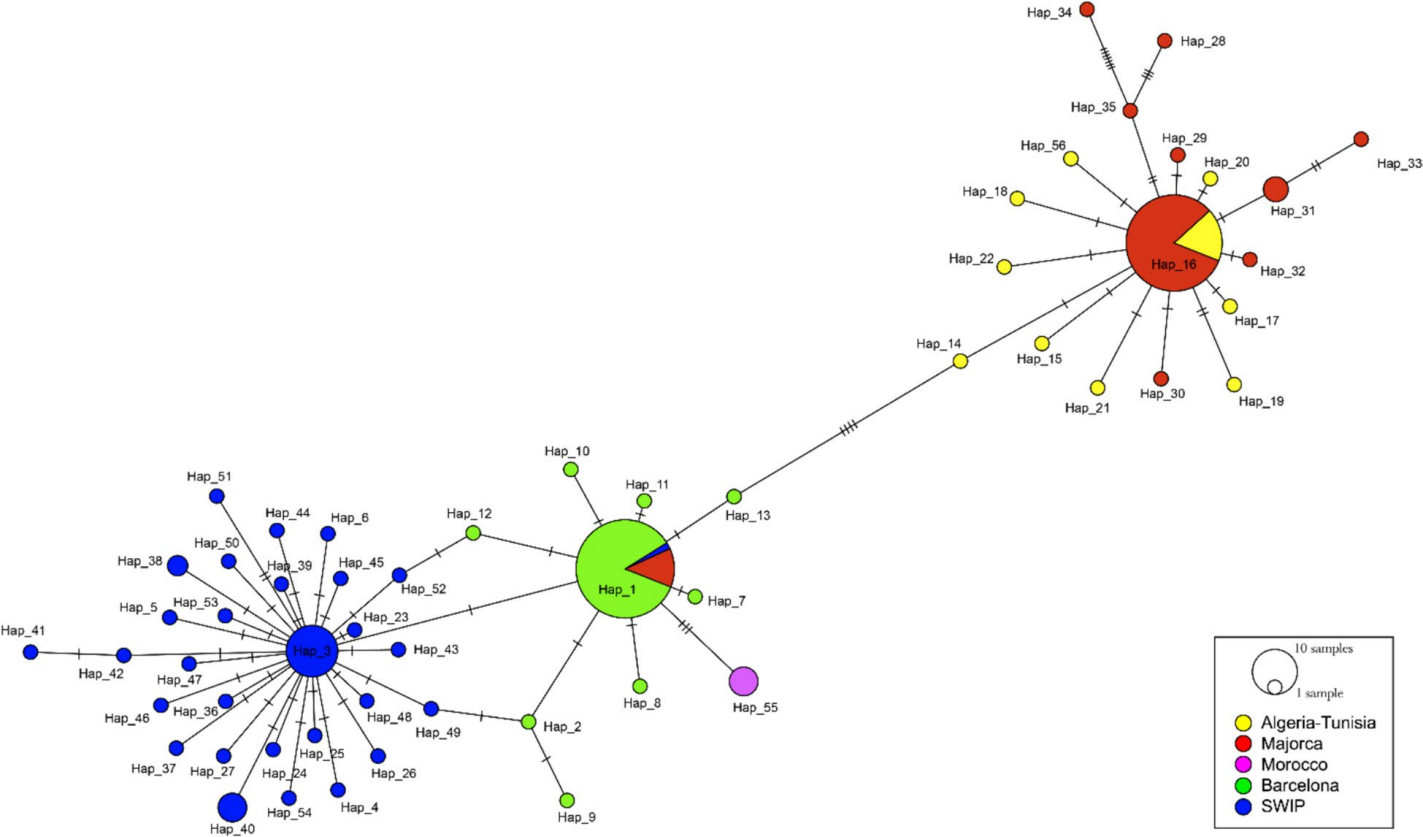
TCS network of *P. perniciosus* COI gene sequences from the present study (Majorca and Barcelona) and from the South and West Iberian Peninsula (SWIP), Algeria–Tunisia, and Morocco retrieved from Genbank. Hatch marks indicate mutations, and circle sizes are proportional to the number of specimens sharing each haplotype

The haplotype parsimony network (TCS) based on COI sequences of *P. perniciosus* revealed a star-like phylogeny comprising 56 haplotypes (Fig. 2). The Majorcan population contained ten haplotypes (Hap1, Hap16, and Hap28 to Hap35), while Barcelona harbored nine (Hap1, Hap2, and Hap7 to Hap13). Hap16 was the most common in Majorca (*n* = 37) and was also detected in the Algeria–Tunisia group (*n* = 8), whose population shared haplotypes exclusively with Majorca. Hap1 was the most frequent overall and predominant in Barcelona (*n* = 40), occurring also in Majorca (*n* = 6) and one specimen from southern Spain. Hap3, the most frequent haplotype in the SWIP, was closely related to Barcelona haplotypes in the TCS network.

Of the 56 haplotypes identified, 54 (96.4%) were private, indicating high genetic differentiation and limited haplotype sharing between populations. Eight private haplotypes were identified in Majorca (Hap28 to Hap35), and eight were also recorded in Barcelona (Hap2, Hap7 to Hap13).

## Discussion

This study used the mitochondrial COI gene to confirm the morphological identification of sand fly species and unravel the genetic relationships between several *P. perniciosus* populations from the Mediterranean basin. The COI marker has proven highly effective for sand fly species identification, particularly when morphological traits are ambiguous [30, 31]. Its relatively rapid mutation rate, high sequence polymorphism, and maternal inheritance also make it a robust tool for phylogeographic studies [22, 50].

The observed genetic structure of *P. perniciosus* in the western Mediterranean suggests that historical and natural barriers have played a key role in driving population divergence. Given the limited active dispersal capabilities of *Phlebotomus* sand flies, which under natural conditions rarely move far from their breeding and resting sites [1, 51], even moderate physical barriers can significantly restrict gene flow and promote genetic differentiation over time [52, 53].

Among potential barriers, marine boundaries are particularly relevant in the Mediterranean, where islands such as Majorca are separated from the mainland by relatively short but ecologically meaningful distances. Although the straight-line distance between Majorca and Barcelona is only approximately 200 km, the Balearic Sea appears to act as a substantial isolating factor for *P. perniciosus*, as evidenced by the significant genetic differentiation observed in this study. The high pairwise *Fst* values between Majorca and Barcelona indicate strong population structure and limited gene flow, consistent with the low *Nm* estimates obtained (< 1). Similar levels of differentiation have been reported in other sand fly populations separated by geographic barriers such as mountain ranges or ecological discontinuities [26, 54]. This pattern was further corroborated by our AMOVA results, which attributed most genetic variation to differences among groups, reflecting a strong overall genetic structure.

Although research has focused primarily on terrestrial barriers, evidence shows that seas can also shape the evolutionary history and distribution of phlebotomine sand flies. For instance, Cruaud et al. [55] demonstrated the role of marine and geological barriers in the diversification of *Paraphlebotomus* species in the Mediterranean region, and Tsirigotakis et al. [56] reported distinct sand fly communities across the Aegean islands. In our study, the genetic divergence between Majorca and Barcelona likely reflects long-term isolation imposed by the Balearic Sea, reinforced by the limited dispersal capacity of *P. perniciosus*.

In contrast, the moderate genetic differentiation observed between Barcelona and the SWIP group suggests more frequent or recent gene flow within the mainland. The limited presence of major topographic barriers and the continuity of suitable habitats may facilitate connectivity across the Iberian Peninsula. Aransay et al. [57] likewise proposed that gene flow among *P. perniciosus* populations can be maintained across large continental areas with few dispersal constraints. Although all *P. perniciosus* specimens in their study belonged to the Iberian lineage, they identified two populations (North and South), consistent with the present findings. Similar patterns have been observed in other vector species: for example, *P. ariasi* in the Iberian Peninsula and *L. anduzei* and *L. umbratilis* in Brazil exhibited moderate genetic structuring among mainland populations, with substantial gene flow within regions [58, 59].

In addition to physical environmental factors, historical processes likely contributed to the observed genetic patterns. During the Last Glacial Maximum, much of Europe was unsuitable for thermophilic taxa such as sand flies, with southern Iberia and North Africa serving as refugia and potential sources for postglacial recolonization [60, 61]. The genetic affinities between Majorcan and Algerian populations, evidenced by shared haplotypes and clustering in phylogenetic and haplotype network analyses (Figs. 1 and 2), may reflect ancient dispersal events across the western Mediterranean. These could have occurred via passive dispersal, human-mediated movement, or postglacial colonization. The presence of *P. perfiliewi* in Majorca, clustering with Algerian lineages [16], further supports the hypothesis of historical connectivity across the Mediterranean. While Pleistocene glacial refugia are frequently cited as the main drivers of lineage diversification in *Phlebotomus* species, Pleistocene sea-level fluctuations may also have intermittently modulated connectivity across the Strait of Gibraltar and shaped gene flow between North Africa and Iberia [62]. At a much deeper timescale, major ancient environmental changes such as the Messinian Salinity Crisis profoundly altered Mediterranean biogeography [55, 63], although their relevance to intraspecific divergence in *Phlebotomus* remains highly uncertain. Moreover, the absence of a significant isolation-by-distance pattern, as indicated by the Mantel test, suggests that geographic distance alone does not explain the marked genetic differentiation observed among populations. This further supports the interpretation that historical and ecological barriers have played a predominant role in shaping population structure.

Intra-population diversity metrics provide further insight into the demographic histories of these populations. Classical island biogeography and population genetic theory predict reduced genetic diversity in insular populations owing to founder effects, small effective population sizes, and restricted gene flow [64, 65]. However, our data reveal slightly higher haplotype and nucleotide diversity in Majorca compared with Barcelona. This suggests a more complex demographic history for the island population, possibly involving multiple colonization events, admixture with divergent lineages, or a relatively large founding population. Phylogenetic and haplotype network analyses support this interpretation: Majorcan sequences form a monophyletic group and occupy distinct positions in the TCS network, with restricted haplotype sharing with Barcelona, consistent with historical isolation and potentially divergent evolutionary trajectories.

In the island of Sri Lanka, *P. argentipes* populations also exhibit high genetic diversity despite geographic isolation [29, 66]. In plants, García-Verdugo et al. [67] showed that insular populations can maintain or exceed mainland genetic diversity under favorable colonization, dispersal, and ecological conditions. Similarly, Sun and Vargas-Mendoza [68] reported complex genetic patterns in *Kleinia neriifolia* from the Canary Islands, shaped by colonization history and isolation. Our findings in *P. perniciosus* align with this broader perspective: the moderately elevated within-population diversity in Majorca may reflect an early colonization event, possibly followed by periods of isolation that allowed the gradual accumulation of novel mutations. While diverging from classical expectations of island genetic erosion, these results support the idea that islands can serve as dynamic reservoirs of genetic diversity under specific historical and ecological scenarios [69].

## Conclusions

Mitochondrial COI sequences were used to investigate the genetic structure of *P. perniciosus* populations from Majorca and Barcelona, with additional sequences from the South and West Iberian Peninsula and North Africa. COI effectively resolved population structure and historical relationships in this species. The significant genetic differentiation between Majorca and Barcelona populations, together with the clustering of Majorca and Algeria–Tunisia populations, suggests historical connections across the western Mediterranean. Within the island population, moderately high genetic diversity may reflect a more complex demographic history than typically expected in insular contexts. In contrast, patterns observed in the mainland populations show greater genetic continuity. These findings indicate that insular and continental populations of *P. perniciosus* may have followed different evolutionary trajectories and provide a foundation for further phylogeographic studies of this leishmaniasis vector. Population structuring may have potential implications for leishmaniasis surveillance and control, as genetically distinct populations may display differences in vector competence, behavior, or response to interventions. Understanding these patterns can help to refine risk assessments and guide targeted vector surveillance in both insular and mainland settings.

## Supplementary Information

**Additional file 1.**

## Data Availability

All data generated or analyzed during this study are included in this published article and its supplementary information files.
