# Supplementary material for: Genetic diversity of Phlebotomus perniciosus populations between insular and mainland regions in the leishmaniasis-endemic western Mediterranean area
Source: Parasit Vectors. 2026 Jan 22;19:85. doi: 10.1186/s13071-026-07261-z (PMC12911238; doi:10.1186/s13071-026-07261-z)
Supplement: Supplementary file 1 — Additional file 1. [file 13071_2026_7261_MOESM1_ESM.docx]

Supplementary Table 1. Phlebotomus perniciosus COI sequences retrieved from GenBank and used in the genetic analyses of this study.

| **Accession number** | **Geographical origin** | **Reference** |
| --- | --- | --- |
| AB985696, AB985700/01/03/07/09/17 | Portugal | [71] |
| OL364762/69/80/97, OL364802/06/07/16 – 20/29/34/37/39 | Portugal | [75] |
| MH559445/54/62/64/77/79/82 | Portugal | Unpublished |
| KJ481136 – 40/42/43/45/46/48/50/51/54 | Algeria | Unpublished |
| OL814952/53/54/55 | Tunisia | [73] |
| LC090044 | Spain | Unpublished |
| OP824886 | Spain | [16] |
| OR076129/40/42/51/60/62/82/83/89, OR076213/25/48/49/67 | Spain | Unpublished |
| MT233398/99/400/401 | Morocco | [74] |

Supplementary Table 2. Number of migrants per generation (*Nm*) estimated from pairwise *Fst* values among *P. perniciosus* populations from the studied geographical origins based on COI gene sequences.

| **Populations** | ***Fst*** | ***Nm*** |
| --- | --- | --- |
| Majorca – Barcelona | 0.78262 | 0.14 |
| Majorca – SWIP | 0.75375 | 0.16 |
| Majorca – Algeria–Tunisia | 0.02470 | 19.74 |
| Majorca – Morocco | 0.73800 | 0.18 |
| Barcelona – SWIP | 0.51225 | 0.48 |
| Barcelona – Algeria–Tunisia | 0.91029 | 0.05 |
| Barcelona – Morocco | 0.89395 | 0.06 |
| SWIP – Algeria–Tunisia | 0.82800 | 0.10 |
| SWIP – Morocco | 0.73030 | 0.18 |
| Algeria–Tunisia –Morocco | 0.87415 | 0.07 |

SWIP: South and West Iberian Peninsula.
